# Effects of Mare Age and Number of Foalings on Body Morphometry, Udder Morphology, Milk Yield, and Milk Composition in Kushum Mares

**DOI:** 10.3390/ani16182864

**Published:** 2026-09-11

**Authors:** Maxat Toishimanov, Gulmira Bekova, Dastanbek Baimukanov, Dinara Begaliyeva, Anastasiya Yanina, Rakhim Kanat, Dias Daurov, Ainash Daurova, Zagipa Sapakhova, Gemingguli Muhatai, Zhaniya Assanbek, Alzhan Shamshidin, Indira Beishova, Malika Shamekova

**Affiliations:** 1 Laboratory of Breeding and Biotechnology, Institute of Plant Biology and Biotechnology, Timiryazev 45, Almaty 050040, Kazakhstan; m.toishmanov@ipbb.kz (M.T.); dbaimukanov@mail.ru (D.B.); d.begaliyeva@ipbb.kz (D.B.); r.kanat@ipbb.kz (R.K.); m.shamekova@ipbb.kz (M.S.); 2 Testing Center, Zhangir Khan West-Kazakhstan Agrarian Technical University, Zhangir Khan 51, Oral 090009, Kazakhstan; 270180as@gmail.com (A.S.); indirabeishova85@gmail.com (I.B.); 3 Tanir Research Laboratory, Al-Farabi Avenue 75B, Almaty 050060, Kazakhstan; 4 College of Animal Science and Technology, Tarim University, Alar 843300, China; gmgl-113@foxmail.com; 5 College of Life Science and Technology, Tarim University, Alar 843300, China; zhaniya.2401a@gmail.com

**Keywords:** Kushum mare, mare milk, body morphometry, udder morphology, milk composition, milk yield, number of foalings, principal component analysis, Pearson correlation

## Abstract

Mare’s milk is recognized for its nutritional value, but information on the relationships of mare age and number of foalings with body conformation, udder morphology, milk yield, and milk composition in the indigenous Kushum breed remains limited. Forty-one clinically healthy lactating Kushum mares were evaluated in June and classified by age (4–7, 8–11, and 12–16 years) and number of foalings (1–3, 4–7, and 8–11). Body traits were measured once; udder morphometry was available for 30 mares. Milk yield was recorded at 08:00, 10:00, 12:00, 14:00, and 16:00 h on two consecutive days. On Day 2, a 100-mL aliquot was collected after each milking, and the five aliquots were pooled into one 500-mL composite sample per mare; physicochemical composition was available for 40 mares. Age and number-of-foalings effects were evaluated in separate models. Continuous morphometric and physicochemical traits were analyzed by one-way ANOVA with Tukey-adjusted comparisons, with Benjamini–Hochberg false discovery rate (FDR) correction across trait families; two-day milk yield was additionally evaluated with a repeated-measures mixed model. After FDR correction, cannon bone circumference, body weight, udder length, and udder width differed among both age and number-of-foalings groups. Teat shape also differed among groups. The two-day mean daily milk yield did not differ by age (*p* = 0.483) or number of foalings (*p* = 0.195), although significant group-by-day interactions were detected. In pooled Day-2 milk, SNF, lactose, glucose, freezing point, acidity, lactic acid, density, and urea differed among both classification factors after FDR correction (q < 0.05), whereas fat, protein, total solids, galactose, citric acid, and casein did not. PCA explained 60.9% of the variance in body/udder traits plus mean milk yield and 68.8% of the variance in physicochemical composition. These findings indicate selective associations of age and number of foalings with Kushum mare phenotype and milk composition, while emphasizing the limitations of small udder subgroups and equal-volume pooled sampling.

## Simple Summary

Kazakh mare’s milk is a valuable traditional food with high nutritional and functional properties. However, information on how mare age and the number of foalings influence body conformation, udder morphology, milk production, and milk composition in the indigenous Kushum breed is limited. This study evaluated 41 lactating Kushum mares and examined body measurements, udder morphometric characteristics, daily milk yield, and physicochemical composition of milk. Body size traits were generally similar among age and foaling groups, whereas several udder measurements, including udder length, udder width, and teat base circumference, varied significantly. Milk yield remained relatively stable regardless of mare age or number of foalings. In contrast, milk composition was affected by both factors, with significant differences observed in solids-not-fat, lactose, glucose, freezing point, acidity, density, and urea concentration, while fat, protein, total solids, and casein remained unchanged. Correlation analysis and principal component analysis revealed strong relationships among udder morphometric traits and among the major milk constituents, whereas daily milk yield was more closely associated with udder morphology than with overall body size. Our results provide new information on phenotypic variation in Kushum mares and may contribute to improved breeding, herd management, and sustainable production of mare’s milk in Kazakhstan.

## 1. Introduction

Mare milk has attracted growing scientific and commercial attention worldwide as a functional food with a nutritional profile that closely resembles that of human milk [1,2]. It has been estimated that mare milk is regularly consumed by approximately 30 million people worldwide, principally across the steppe regions of Central Asia, Mongolia, China, and parts of Eastern Europe [3,4]. Compared with bovine milk, equine milk is characterized by a lower fat and casein content, a higher lactose concentration, and a near 1:1 casein-to-whey protein ratio, features that render it easily digestible and suitable for infants with cow’s milk protein allergy [1,5,6]. In Central Asia, most mare milk is consumed after lactic-alcoholic fermentation as the traditional beverage koumiss (qymyz), a product of substantial cultural, nutritional, and economic importance whose fermentation generates additional bioactive peptides and probiotic microorganisms [4,7,8]. This combination of nutritional and therapeutic attributes underlies the increasing interest in mare milk within the expanding functional-food and nutraceutical markets [2,9].

The yield and composition of mare milk are governed by a complex interaction of genetic and environmental factors. Breed and body size are major determinants, with heavy and dairy breeds generally producing more milk than lighter saddle breeds [3,10]. Stage of lactation exerts a strong influence: fat and protein concentrations typically decline while lactose increases as lactation advances, and total yield follows a characteristic curve that peaks between roughly 20 and 90 days depending on breed [10,11,12]. Mare age and number of foalings also modify both production and composition, with reported effects on protein, sodium, and fatty acid profiles, although these factors have been studied far less than lactation stage and remain incompletely understood [13,14]. Feeding regimen and pasture type, body condition at foaling, environmental conditions, milking method, and overall management further contribute to the pronounced multifactorial variability observed in equine milk, particularly in the fat fraction [14,15,16,17]. Because the cisternal compartment of the mare udder is small and most milk is held in the alveolar fraction, milk ejection is strongly dependent on oxytocin release and on adequate conditioning to the milking routine, making management practices especially influential in dairy mare systems [17,18].

Body morphometry and udder morphology are widely used in dairy science as practical, low-cost indicators of productive potential and as selection criteria in breeding programs. In horses, linear body measurements such as height at the withers, body length, and chest girth are routinely used to characterize breeds, estimate live weight, and assess conformation in relation to function and productivity [19,20,21]. In dairy ruminants, numerous studies have demonstrated positive phenotypic and genetic correlations between udder and teat dimensions and milk yield: in zebu cows of northern Cameroon, udder diameter (r = 0.541) and udder height (r = 0.549) were significantly correlated (*p* < 0.001) with milk yield [22], and in Friesian cows udder width and rear udder depth were identified as good predictors of milk yield and lactation length, with udder width showing strongly positive genetic correlations with total yield (0.86) and persistency (0.93) [23]. Similar relationships have been reported in dairy goats, where udder volume and globularity correlate more strongly with yield and milking ability than length measurements [24,25], and in camels [26]. Comparable work in mares, though scarcer, indicates that udder shape and measurements are related to milk productivity and adaptability to machine milking [27,28]. These findings support the use of body and udder measurements as tools for identifying superior dairy individuals across species.

The physicochemical composition of mare milk has been described in several breeds, yet reported values vary considerably. Average fat content generally ranges from below 1% to about 2%, protein from roughly 1.3% to 2.3%, lactose from about 5.7% to 6.9%, and solids-not-fat around 9% [1,5,29,30]. Studies on Lipizzaner mares reported mean values of 1.18% fat, 1.53% protein, 6.56% lactose, and 9.01% solids-not-fat [30], whereas Haflinger and other breeds have shown differing casein contents and nitrogen fractions [31,32]. Physical properties such as density, titratable acidity, freezing point, and pH also fluctuate with lactation stage and diet, and minor constituents including casein, urea, and citric acid are increasingly measured with infrared analyzers calibrated for equine milk [13,29,33]. The inconsistencies among published studies—arising from differences in breed, management, sampling and milking protocols, lactation stage, and analytical methods—complicate direct comparisons and highlight the need for standardized, breed-specific characterization [13,14,16].

Mare-milk production is geographically concentrated and differs substantially from conventional dairy systems. It has a long-established role in Kazakhstan and other Central Asian countries, where mares are traditionally milked for the production of fermented products such as kumys, and mare-milk production is also practiced in parts of Russia and Europe. In contrast, specialized mare-dairy production remains comparatively limited in the Americas and has not developed into a large-scale dairy sector comparable with those based on cattle, sheep, or goats. This geographical concentration highlights the importance of characterizing the productive and morphological traits of local dairy horse populations and identifying animal-related factors associated with milk yield and composition [34,35,36,37].

Although individual factors influencing mare milk have each received attention, few studies have simultaneously integrated body morphometry, udder morphology, milk yield, and physicochemical milk composition while accounting for mare age and number of foalings, and such combined data are almost entirely lacking for the Kushum breed under natural pasture conditions. Recent work on other Kazakh mares has begun to address the effects of age, number of foalings, and lactation stage on milk composition using multivariate approaches, but the interrelationships among conformation, udder traits, yield, and composition remain largely unexplored. Therefore, the aim of this study was to evaluate the effects of mare age and number of foalings on body morphometry, udder morphology, milk yield, and physicochemical composition of milk in Kushum mares, and to investigate the relationships among these traits using correlation analysis and principal component analysis.

## 2. Materials and Methods

### 2.1. Study Area and Animals

The study was conducted in the West Kazakhstan Region, Kazakhstan, where horse breeding is traditionally based on an extensive pasture-based management system. The area has a strongly continental and arid climate, and natural steppe and semi-desert pastures constitute the principal forage resource for grazing livestock. During the study period, the mares were maintained under traditional pasture-based conditions and grazed natural pastures typical of the region. The pasture vegetation is predominantly composed of grass–forb and grass–Artemisia communities, with feather grasses (*Stipa* spp.), fescue (*Festuca* spp.), wheatgrasses (*Agropyron* spp.), and wormwoods (*Artemisia* spp.) representing characteristic forage plants of these rangelands.

A total of 41 clinically healthy lactating Kushum mares were included in the study. All mares were maintained under identical husbandry and feeding conditions and grazed on natural pasture throughout the experimental period. Fresh drinking water was available ad libitum. The animals were managed according to the routine management practices of the farm, and no dietary supplements or medications that could influence milk production or composition were administered during the study.

All mares were examined in June during the same field-observation period to reduce variation associated with season and stage of lactation. The animals were classified according to mare age into three categories: 4–7 years (*n* = 14), 8–11 years (*n* = 12), and 12–16 years (*n* = 15). The same mares were also classified according to the number of previous foalings into three groups: G1, 1–3 foalings (*n* = 13); G2, 4–7 foalings (*n* = 14); and G3, 8–11 foalings (*n* = 14).

The age of each mare and the number of previous foalings were obtained from the official breeding records maintained by the farm. Only healthy mares with complete production records and without visible signs of mastitis, lameness, metabolic disorders, or other clinical diseases were included in the study.

This observational design allowed associations of mare age and number of foalings with body morphometry, udder morphology, milk yield, and milk physicochemical composition to be evaluated under the same management and seasonal conditions. Because age and number of foalings were closely related biological characteristics, they were analyzed in separate statistical models.

### 2.2. Experimental Design

Body morphometric measurements included height at the withers, oblique body length, chest girth, cannon bone circumference, and body weight. Udder morphometric measurements included udder length, udder width, udder depth, udder base circumference, teat length, teat base circumference, and distance between teats. Qualitative udder traits included udder shape, teat shape, and milk vein development.

Milk yield was recorded at five consecutive milkings (08:00, 10:00, 12:00, 14:00, and 16:00 h) on each of two consecutive days. Daily milk yield was calculated as the sum of the five milkings, and the mean of the two daily totals was used as the representative milk-yield value for each mare.

For physicochemical analysis, milk was sampled on Day 2 only. After thorough mixing of the milk obtained at each milking, a 100-mL aliquot was collected. The five equal-volume aliquots (5 × 100 mL) were combined to obtain one 500-mL pooled composite sample per mare. The pooled samples were immediately frozen in a portable freezer and maintained frozen until laboratory analysis. Thus, physicochemical composition was determined from one equal-volume pooled Day-2 sample per mare, rather than from five independent samples. The pooled sample incorporated milk from all five daytime milkings but was not weighted according to the milk volume obtained at each milking.

### 2.3. Body and Udder Morphometric Measurements

Body morphometric measurements were obtained from all 41 lactating Kushum mares once, immediately before the first morning milking at 08:00 h, using standard zootechnical procedures. All measurements were performed by the same trained operator to minimize observer variation. Linear measurements were recorded to the nearest 0.1 cm using a flexible measuring tape or a measuring stick, whereas body weight was determined using an electronic livestock scale and recorded to the nearest 1 kg.

The following body measurements were recorded: height at the withers (cm), measured as the vertical distance from the highest point of the withers to the ground; oblique body length (cm), measured from the point of the shoulder to the point of the buttock; chest girth (cm), measured immediately behind the forelimbs around the thorax; cannon bone circumference (cm), measured at the midpoint of the left fore cannon bone; and body weight (kg).

Udder morphometric measurements were performed once for each available mare immediately before the first morning milking at 08:00 h to ensure comparable udder filling. The following traits were measured: udder length, udder width, udder depth, udder base circumference, teat length, teat base circumference, and distance between teats, using the anatomical definitions described above. The anatomical landmarks and locations used for the body and udder morphometric measurements are illustrated in Figure S1.

### 2.4. Udder Morphology Assessment

In addition to quantitative morphometric measurements, qualitative udder characteristics were evaluated once in all 41 lactating Kushum mares before the first morning milking at 08:00 h by visual inspection and palpation. All assessments were performed by the same experienced evaluator to ensure consistency throughout the study.

Udder morphology was classified according to three qualitative characteristics: udder shape, teat shape, and mammary vein development [38,39,40].

Based on external appearance, udders were categorized as bath-shaped, bowl-shaped, or round. Bath-shaped udders were characterized by an elongated and relatively shallow appearance with a broad attachment to the abdominal wall. Bowl-shaped udders exhibited a symmetrical hemispherical shape with moderate depth and well-developed glandular tissue, whereas round udders were compact and nearly spherical in appearance.

Teat morphology was classified into two categories according to teat conformation: cylindrical and conical. Cylindrical teats maintained a relatively uniform diameter along their entire length, whereas conical teats gradually tapered from the base toward the teat end.

Mammary vein development was assessed visually using criteria adapted from previously published morphological evaluation approaches for dairy mares and the official horse-grading guidelines of the Republic of Kazakhstan [39,40]. The assessment was based on the visibility, prominence, and degree of development of the mammary veins. For the present study, mammary vein development was classified into three categories: poor, characterized by thin or barely visible veins; moderate, characterized by clearly visible veins of intermediate development; and well developed, characterized by prominent, enlarged, and readily distinguishable veins on the ventral abdominal surface. The same criteria were applied consistently to all mares.

### 2.5. Milk Collection and Milk Yield Determination

Milk yield was recorded using the routine manual-milking protocol applied to lactating Kushum mares. Before each milking, foals were temporarily separated from their dams for approximately 2 h. Immediately before milking, each foal was allowed to suckle briefly to stimulate milk let-down, after which the foal was removed and the mare was milked manually.

Milk collection was performed five times daily at 08:00, 10:00, 12:00, 14:00, and 16:00 h on two consecutive observation days. At each milking, the total harvestable milk from both udder halves was collected into a graduated container, and the volume was recorded to the nearest 0.01 L. This five-times-daily milking schedule, with approximately 2-h intervals between milkings, reflected the routine management practice for lactating mares at the study farm during the milk-production period and was not imposed specifically for the experiment. The relatively frequent milking schedule was used because of the limited milk-storage capacity of the equine udder and the need for regular milk removal during daytime mare-milk production. The same milking schedule was maintained on both observation days to ensure standardized assessment of daily milk yield.

Milk for physicochemical analysis was collected only on Day 2. After each milking, the milk was thoroughly mixed, and a 100-mL aliquot was transferred to the mare-specific composite container. After the fifth milking, the five aliquots were combined to yield a 500-mL equal-volume pooled sample. The pooled sample was immediately frozen in a portable freezer and maintained frozen until laboratory analysis.

### 2.6. Physicochemical Characteristics and Composition of Milk

Prior to analysis, the frozen pooled samples were thawed under controlled conditions and thoroughly homogenized. Each pooled sample represented equal-volume aliquots collected from the five milkings of the second observation day. Milk samples were thoroughly mixed immediately before analysis. The physicochemical composition of the milk, including fat, protein, solids-not-fat (SNF), total solids (TS), lactose, galactose, glucose, freezing point, titratable acidity, lactic acid, density, citric acid, urea, and casein, was determined using an automated MilkoScan FT2 milk analyzer (Foss Electric A/S, Hillerød, Denmark) based on Fourier transform infrared (FTIR) spectroscopy.

Because the standard factory calibration of the instrument is primarily designed for bovine milk, a mare milk-specific calibration was established prior to analysis. Calibration was developed using reference mare milk samples with known chemical composition previously determined by validated reference methods, including the Kjeldahl method for protein and the Gerber method for fat. The calibration model was subsequently validated by comparing FTIR-predicted values with the corresponding laboratory reference values, confirming the suitability of the customized calibration for the quantitative analysis of mare milk.

All pooled milk samples were analyzed under identical operating conditions. Each composite sample was analyzed in duplicate as a technical analytical replicate, and the mean of the duplicate instrument readings was retained as the single physicochemical value for that mare. Quality control included routine instrument calibration and analysis of reference control samples throughout the analytical period.

### 2.7. Statistical Analysis

Statistical analyses were performed using JMP Pro version 18.0 (SAS Institute Inc., Cary, NC, USA) and verified during revision with Python 3.13 using SciPy 1.17.0 and statsmodels 0.14.6. Continuous variables were examined for distributional assumptions before inferential analysis. Because mare age and number of foalings were closely related, separate models were fitted for the two classification factors.

Body and udder morphometric traits and pooled milk physicochemical characteristics were analyzed using separate one-way ANOVA models for mare age and number of foalings. When an overall group effect was significant, Tukey-adjusted pairwise comparisons were performed. Results are presented as mean ± standard error (SE). To account for multiple testing, Benjamini–Hochberg false discovery rate (FDR) correction was applied separately across the morphometric trait family and across the 14 physicochemical traits for each classification factor; raw *p*-values and FDR-adjusted q-values are reported.

Associations between categorical udder characteristics (udder shape, teat shape, and milk vein development) and age or number-of-foalings groups were evaluated using the Fisher–Freeman–Halton exact test. FDR adjustment was additionally applied across the three categorical udder traits within each classification factor.

Milk yield recorded on Day 1 and Day 2 was evaluated with separate linear mixed-effects models for mare age and number of foalings, with day as the within-mare repeated factor and mare as a random intercept. Milk-yield data were analyzed using linear mixed-effects models to account for repeated observations of the same mare on two consecutive days. Mare was included as a random effect, while observation day and either mare age group or number-of-foalings group, together with their interaction, were included as fixed effects. Separate models were fitted for mare age and number of foalings. The two-day mean daily yield was also summarized for each mare and compared among classification groups. Pearson correlations were calculated using pairwise available observations and visualized as a heatmap. Body and udder morphometric traits and physicochemical milk characteristics were analyzed using one-way ANOVA separately according to mare age and number of foalings. For physicochemical characteristics, repeated-measures analysis was not applicable because the five Day-2 aliquots were combined before analysis to obtain one pooled composite sample per mare. Tukey’s HSD test was used for post hoc comparisons when appropriate, and the Benjamini–Hochberg procedure was applied to control the FDR.

Principal component analysis (PCA) was performed on standardized variables. Two PCA models were constructed: body and udder morphometric traits together with the two-day mean daily milk yield and Day-2 pooled milk physicochemical characteristics. Mare age group was used only to visualize score distributions and was not included as a PCA input variable. The first two principal components were retained for biplot visualization.

Statistical significance was evaluated at *p* < 0.05, with FDR-adjusted q < 0.05 used to identify findings that remained significant after correction for multiple testing.

For all statistical analyses, differences were considered statistically significant at *p* < 0.05.

## 3. Results

### 3.1. Morphometric Characteristics of Kushum Mares

The body and udder morphometric characteristics according to mare age and number of foalings are presented separately in Table 1 and Table 2, respectively.

Cannon bone circumference differed significantly among age groups (*p* < 0.001, q = 0.003). Mares aged 8–11 years had the greatest mean cannon bone circumference (20.12 ± 0.16 cm), compared with mares aged 4–7 years (19.04 ± 0.21 cm) and 12–16 years (19.15 ± 0.19 cm). Body weight also differed among age groups (*p* = 0.007, q = 0.022), with the highest value observed in mares aged 4–7 years (489.93 ± 16.59 kg), followed by mares aged 8–11 years (450.83 ± 11.13 kg) and 12–16 years (431.27 ± 9.77 kg).

Udder length showed a marked decrease with advancing age (*p* < 0.001, q = 0.002), declining from 26.33 ± 1.20 cm in mares aged 4–7 years to 24.17 ± 1.02 cm in the 8–11-year group and 19.60 ± 0.57 cm in mares aged 12–16 years. A similar age-related pattern was observed for udder width (*p* < 0.001, q = 0.002), with mean values of 20.33 ± 0.33, 19.17 ± 0.66, and 16.53 ± 0.32 cm in the 4–7-, 8–11-, and 12–16-year groups, respectively.

Height at the withers, oblique body length, chest girth, udder depth, udder base circumference, and teat length did not differ significantly among age groups after FDR correction (q > 0.05). Teat base circumference showed a nominal age-associated difference (*p* = 0.023), increasing from 8.17 ± 0.44 cm in the youngest mares to 10.17 ± 0.31 cm in the oldest group; however, this association did not remain significant after multiple-testing correction (q = 0.056). Likewise, the distance between teats tended to decrease with age, from 7.33 ± 0.88 cm in mares aged 4–7 years to 6.40 ± 0.21 cm in mares aged 12–16 years (*p* = 0.046), but this difference was not significant after FDR adjustment (q = 0.092)

Cannon bone circumference differed significantly among groups (*p* = 0.010, q = 0.031). The greatest mean value was observed in mares with 4–7 foalings (19.92 ± 0.20 cm), compared with mares with 1–3 foalings (19.05 ± 0.22 cm) and those with 8–11 foalings (19.19 ± 0.20 cm). Body weight also differed significantly according to number of foalings (*p* = 0.002, q = 0.013). Mares with 1–3 foalings had the highest mean body weight (496.46 ± 16.48 kg), followed by mares with 4–7 foalings (444.64 ± 10.37 kg) and 8–11 foalings (432.79 ± 10.36 kg).

Marked differences were also observed in udder dimensions. Udder length decreased progressively across foaling-number groups, from 27.50 ± 0.50 cm in mares with 1–3 foalings to 23.64 ± 1.01 cm in mares with 4–7 foalings and 19.79 ± 0.57 cm in mares with 8–11 foalings (*p* < 0.001, q = 0.011). A similar pattern was observed for udder width, with corresponding values of 20.50 ± 0.50, 18.93 ± 0.64, and 16.64 ± 0.33 cm, respectively (*p* = 0.003, q = 0.013).

Height at the withers, oblique body length, chest girth, udder depth, udder base circumference, teat length, and teat base circumference did not differ significantly among foaling-number groups after FDR correction (q > 0.05). The distance between teats decreased numerically from 7.50 ± 1.50 cm in mares with 1–3 foalings to 6.36 ± 0.22 cm in mares with 8–11 foalings and showed a nominal group difference (*p* = 0.035); however, this association did not remain significant after FDR adjustment (q = 0.083).

### 3.2. Udder Morphology Characteristics

The distribution of udder shape, teat shape, and milk vein development according to mare age and number of foalings is presented in Table 3.

Udder shape was not significantly associated with either mare age (*p* = 0.107, q = 0.161) or number of foalings (*p* = 0.171, q = 0.257). Bowl-shaped udders were the most frequent type in all groups, accounting for 50.0–73.3% of mares across age groups and 57.1–71.4% across foaling-number groups. Round udders were relatively uncommon and occurred mainly in the oldest mares and those with the greatest number of foalings.

Teat shape differed significantly among both age groups (*p* = 0.010, q = 0.030) and foaling-number groups (*p* = 0.005, q = 0.015), with both associations remaining significant after FDR correction. Cylindrical teats were observed in 50.0% of mares aged 4–7 years but in only 8.3% and 6.7% of mares aged 8–11 and 12–16 years, respectively. Correspondingly, the prevalence of conical teats increased from 50.0% in the youngest group to 91.7% and 93.3% in the two older age groups. A similar distribution was observed according to number of foalings: cylindrical teats occurred in 53.8% of mares with 1–3 foalings but in only 7.1% of mares in both the 4–7 and 8–11 foaling groups, whereas conical teats predominated in the latter two groups (92.9% in each).

Milk vein development showed no significant association with either mare age (*p* = 0.628, q = 0.628) or number of foalings (*p* = 0.694, q = 0.694). Moderately developed milk veins represented the most frequent category, occurring in 50.0–73.3% of mares across age groups and 46.2–71.4% across foaling-number groups.

### 3.3. Milk Yield According to Age and Number of Foalings

Milk yield characteristics according to mare age and number of foalings are presented in Table 4.

Among age groups, the two-day mean daily milk yield was highest in mares aged 4–7 years (4.26 ± 0.45 L/day), followed by mares aged 12–16 years (3.87 ± 0.53 L/day) and 8–11 years (3.37 ± 0.52 L/day). On Day 1, mean daily milk yield ranged from 3.44 ± 0.54 to 3.93 ± 0.55 L/day, whereas on Day 2 it ranged from 3.30 ± 0.51 to 4.63 ± 0.50 L/day.

When mares were classified according to number of foalings, the highest two-day mean daily milk yield was observed in mares with 1–3 foalings (4.46 ± 0.44 L/day), followed by mares with 8–11 foalings (3.98 ± 0.56 L/day), whereas mares with 4–7 foalings had the lowest mean yield (3.18 ± 0.46 L/day). The corresponding Day 1 values were 4.08 ± 0.40, 3.23 ± 0.48, and 4.05 ± 0.57 L/day, while Day 2 values were 4.84 ± 0.49, 3.13 ± 0.45, and 3.91 ± 0.55 L/day for mares with 1–3, 4–7, and 8–11 foalings, respectively.

### 3.4. Milk Physicochemical Composition

The physicochemical composition of milk according to mare age and number of foalings is presented in Table 5 and Table 6.

Milk physicochemical characteristics varied according to both mare age and number of foalings. Among age groups, significant differences after FDR correction were observed for SNF, lactose, glucose, freezing point, acidity, lactic acid, density, and urea (q < 0.05). Mares aged 4–7 years had higher SNF (9.77 ± 0.05%), lactose (7.14 ± 0.05%), density (1042.65 ± 0.32 g/L), and urea (61.48 ± 2.57 mg/dL) than the older groups, together with a more negative freezing point (−0.789 ± 0.007 °C). Glucose was highest in mares aged 12–16 years (0.333 ± 0.014%), while acidity and lactic acid decreased from 10.11 ± 0.21 °D and 0.101 ± 0.002% in the youngest group to 9.34 ± 0.15 °D and 0.093 ± 0.002%, respectively, in the oldest group.

A comparable pattern was observed according to the number of foalings. Significant differences after FDR correction were found for SNF, lactose, glucose, freezing point, acidity, lactic acid, density, and urea (q < 0.05). Mares with 1–3 foalings had higher SNF (9.77 ± 0.05%), lactose (7.14 ± 0.05%), density (1042.65 ± 0.32 g/L), and urea (61.48 ± 2.57 mg/dL), whereas glucose was highest in mares with 8–11 foalings (0.337 ± 0.014%). Acidity and lactic acid declined from 10.11 ± 0.21 °D and 0.101 ± 0.002% in mares with 1–3 foalings to 9.32 ± 0.16 °D and 0.093 ± 0.002% in those with 8–11 foalings. Fat, protein, total solids, galactose, citric acid, and casein did not differ significantly by either age or number of foalings after FDR correction (q > 0.05).

### 3.5. Correlation Analysis

**Figure 1:**
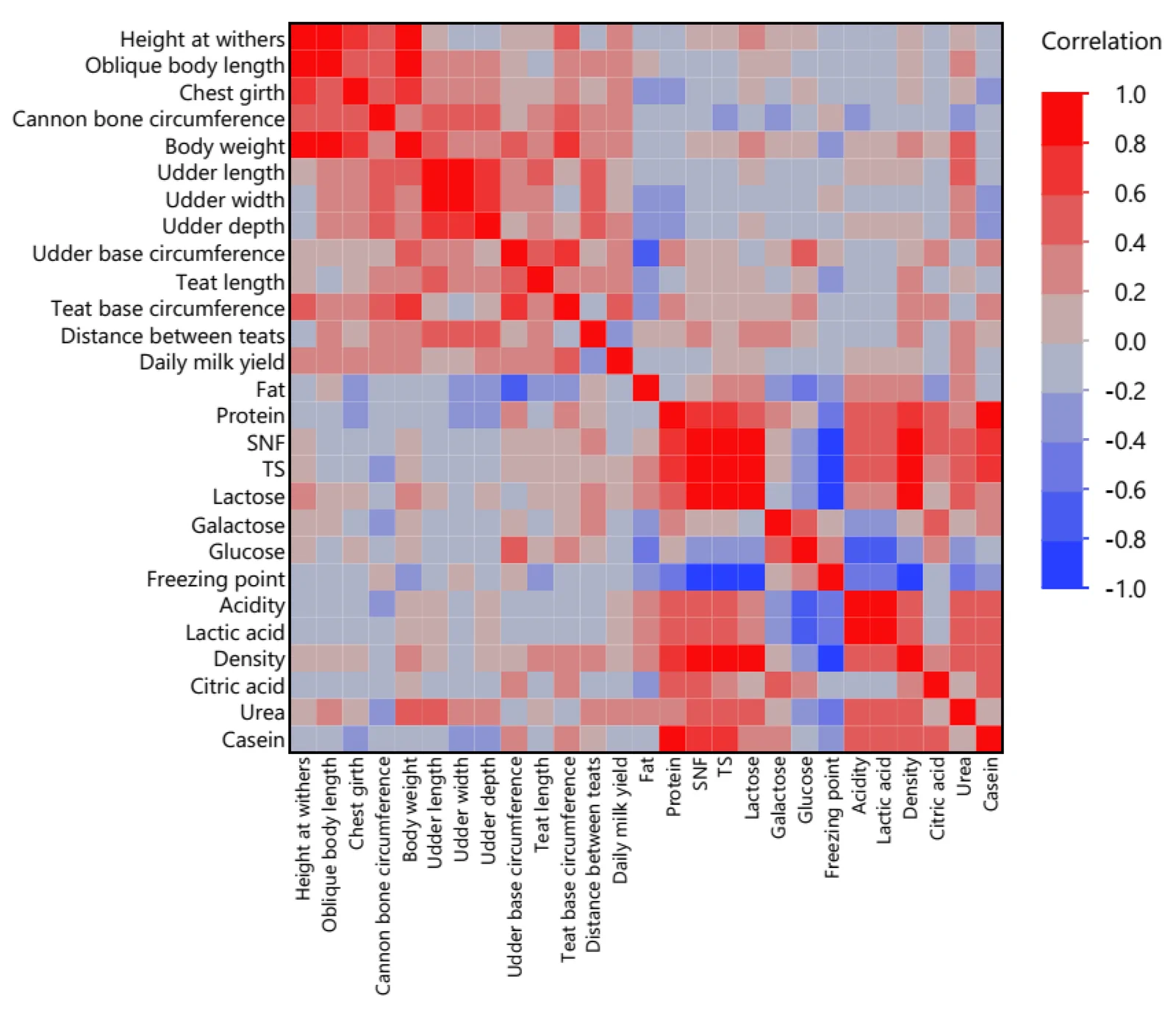
Pearson correlation matrix of body morphometry, udder morphology, daily milk yield, and milk physicochemical characteristics in Kushum mares.

The Pearson correlation matrix describing the relationships among body measurements, udder morphometric traits, milk yield, and milk physicochemical characteristics is presented in Figure 1. Overall, several distinct clusters of correlated variables were identified, reflecting the structural relationships among morphometric and milk quality traits.

Strong positive correlations were observed among the body measurements, including height at the withers, oblique body length, chest girth, cannon bone circumference, and body weight, indicating that larger mares generally exhibited proportional increases in overall body size. Similarly, udder length, udder width, and udder depth were strongly and positively correlated with one another, demonstrating coordinated variation in udder morphology.

Daily milk yield showed positive associations with several udder morphometric traits, particularly teat base circumference and udder dimensions, whereas its relationships with general body measurements were comparatively weaker. Negative correlations were observed between daily milk yield and the distance between teats, suggesting that mares with a shorter distance between teats tended to produce more milk.

Within the milk composition variables, a strong positive correlation cluster was identified among protein, SNF, lactose, density, and casein, indicating that these components varied consistently. Titratable acidity and lactic acid also exhibited a strong positive relationship. In contrast, freezing point showed negative correlations with SNF, lactose, density, acidity, and lactic acid, reflecting the inverse relationship between freezing point depression and milk solids concentration.

Fat content demonstrated relatively weak correlations with most physicochemical variables, whereas glucose showed moderate positive associations with urea and several compositional traits. Citric acid exhibited only weak correlations with the majority of the analyzed variables.

### 3.6. Principal Component Analysis

**Figure 2:**
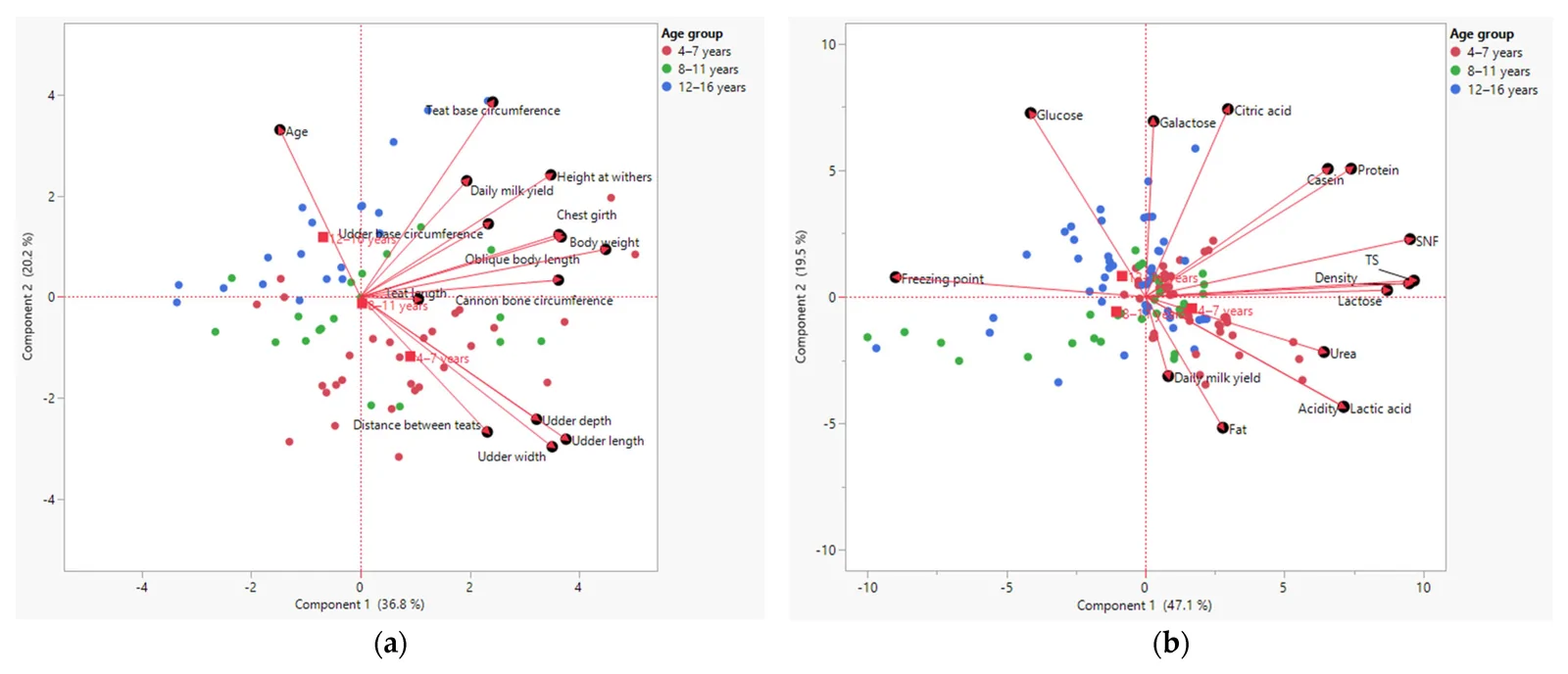
Principal component analysis (PCA) of morphometric traits, milk yield, and milk physicochemical characteristics in Kushum mares according to age groups. (**a**) PCA biplot of body morphometric traits, udder morphometric traits, and daily milk yield. (**b**) PCA biplot of milk physicochemical characteristics and daily milk yield. The arrows represent the loading vectors of the analyzed variables, and the colored symbols indicate mares classified according to age group (4–7, 8–11, and 12–16 years).

The PCA of body and udder morphometric traits together with the two-day mean daily milk yield is presented in Figure 2a. The first two principal components explained 57.0% of the total variance, with PC1 accounting for 36.8% and PC2 for 20.2%.

PC1 was primarily associated with body morphometric variables, including height at the withers, body weight, chest girth, oblique body length, and cannon bone circumference, together with udder length, udder width, and udder depth, indicating that this axis largely represented variation in overall body and udder size. The two-day mean daily milk yield was positively associated with PC1 and was positioned relatively close to height at the withers and teat base circumference. In contrast, the distance between teats was oriented in the opposite direction to milk yield, indicating a negative relationship between these variables.

PC2 was mainly associated with teat base circumference, whereas udder width, udder length, and udder depth showed negative contributions to this component. When mare age groups were superimposed on the PCA score plot for visualization, substantial overlap among the three groups was observed. Mares aged 12–16 years tended to occur more frequently toward the positive side of PC2, whereas younger mares were more frequently distributed toward the lower part of the score plot. However, mare age group was not included as an input variable in the PCA.

The PCA of milk physicochemical characteristics is shown in Figure 2b. The first two principal components explained 66.6% of the total variance, with PC1 and PC2 accounting for 47.1% and 19.5%, respectively.

Protein, casein, SNF, total solids, lactose, and density exhibited positive loadings on PC1 and were positioned relatively close to one another, indicating positive relationships among these milk constituents. Urea, lactic acid, acidity, and fat were also positively associated with PC1, whereas freezing point and glucose were positioned on the opposite side of the axis.

PC2 was mainly associated with glucose, galactose, and citric acid, whereas fat, acidity, and lactic acid showed negative loadings on this component. The distribution of individual mares showed considerable overlap among the three age groups, with no clearly separated clusters, indicating that the multivariate variation in the measured physicochemical characteristics was not strongly separated according to mare age.

## 4. Discussion

The present study provides a phenotypic characterization of lactating Kushum mares, with particular emphasis on the associations of mare age and number of foalings with body and udder morphology, milk yield, and physicochemical milk characteristics. The results indicate that both mare age and reproductive history are associated with specific morphometric and milk-related traits; however, these associations were not uniform across all measured variables. After correction for multiple testing, the most consistent differences in morphometric characteristics were observed for cannon bone circumference, body weight, udder length, and udder width. These findings suggest that age- and reproductive-history-related differences in body development and udder morphology should be considered when evaluating the productive characteristics of Kushum mares. Consistent with our earlier work on Kazakh mares [13,14], the results reinforce the view that individual variation, rather than age class per se, dominates the productive phenotype of these steppe mares.

The near-absence of age and number of foalings effects on height at withers, oblique body length, and chest girth is biologically expected and reflects the fixed nature of the mature equine skeleton rather than any limitation of sampling. Horses attain most measures of somatic and skeletal maturity relatively early, with major body dimensions and skeletal development approaching maturity by approximately 2 years of age [41]. In conventional breeding systems, mares are generally not bred before approximately 3 years of age, and first foaling and the onset of lactation commonly occur at approximately 3–4 years [42]. Thus, although reproductive activity may begin while some aspects of tissue maturation are still continuing, the major phase of structural growth generally precedes or overlaps only partly with the onset of productive life. In the present study, the youngest mares were 4 years old, suggesting that most animals had already completed the major phase of body growth. This may partly explain why several body morphometric traits showed limited variation among age groups. Nevertheless, significant age-associated differences remained for specific traits, including cannon bone circumference, body weight, udder length, and udder width after FDR correction, indicating that some phenotypic characteristics may continue to differ among mature mares. The measured values are consistent with published Kushum breed standards and with the morphometric ranges reported for other working and native horse populations [19,20,21,34]. Against this fixed frame, the significant decline in body weight with advancing age and number of foalings—heaviest in the youngest, lowest-number-of-foalings mares and lowest in the oldest group—is the more informative finding, because weight is a labile, condition-dependent trait rather than a skeletal one. Under natural pasture with no supplementation, the cumulative energetic cost of repeated gestations and lactations must be met largely from body reserves, and the recurrent mobilization of fat and, to a lesser extent, muscle is a plausible driver of the lower mass of older multiparous mares [12,15]. Age-associated declines in dentition and feed-processing capacity may compound this drain in the oldest animals, so that body weight integrates the lifetime reproductive and nutritional history in a way that frame size cannot [19]. The significant effect on cannon bone circumference, peaking in the intermediate group, is best interpreted cautiously as a marker of skeletal robustness and appositional bone deposition rather than of longitudinal growth, since cannon-bone girth can continue to thicken in diameter throughout life in response to mechanical loading even after the bone has stopped lengthening [41]; a mid-life peak may also reflect cohort or selection effects within this single herd and should not be over-interpreted from a cross-sectional sample.

The decrease in udder length and udder width with advancing age and number of foalings is one of the more unexpected outcomes of this study, because it runs opposite to the classic ruminant pattern in which udder dimensions and secretory mass tend to increase across the first several lactations [43]. Several non-exclusive explanations may reconcile this contrast. First, the equine mammary gland is small and largely non-cisternal, storing the great majority of its milk—on the order of 70–85%—in the alveolar compartment rather than in a large cistern, so gross external udder dimensions in the mare are only loosely coupled to secretory capacity and are strongly influenced by the degree of udder fill at the moment of measurement [10,44]. Second, repeated lactations in aged multiparous females are associated with progressive loss of tone in the suspensory apparatus and with partial replacement of secretory parenchyma by connective and adipose tissue; in high-yielding dairy cows, udder and teat conformation demonstrably change across successive lactations, with the udder tending to sag and the distance between fore and rear teats increasing significantly in older cows [43]. Our observation that the distance between teats decreased with age and number of foalings is therefore the reverse of the bovine pattern and, taken with the reduced udder length and width, may indicate a more involuted, less voluminous, and more compact gland in older Kushum mares rather than the pendulous, widely spread udder typical of aged dairy cows. Third, the decline in udder dimensions parallels the decline in body weight, so part of the apparent effect may simply be a correlate of reduced overall body condition. The significant increase in teat base circumference with age—alongside a non-significant trend in the number of foalings (*p* = 0.068) and a non-significant decreasing tendency in udder depth (*p* = 0.061 for age)—is consistent with cumulative mechanical remodeling of the teat and does not contradict a reduction in the gland proper. Because udder morphometry was available for only 41 mares, with very small youngest-age (n = 3) and lowest-number of foalings (n = 2) subgroups, these particular comparisons must be regarded as indicative rather than definitive, and comparisons with dairy cows, goats, sheep, and camels [23,24,25,26] should be read in light of the mare’s fundamentally different, alveolar-dominant udder.

The dissociation between teat shape, which changed markedly with both age and number of foalings, and overall udder shape and milk-vein development, which did not, points to teat conformation being an acquired, plastic trait while gross udder form is largely genetically determined and breed-typical. The shift from cylindrical teats in the youngest mares (about 50%) to a strong predominance of conical teats in older, higher-number-of-foalings mares—exceeding 90% in the oldest groups—is most plausibly explained by the repeated mechanical stress imposed on the teat by suckling foals and by hand milking, accumulating across successive lactations and gradually reshaping teat-end and teat-barrel conformation. An analogous number of foalings- and milking-related changes in teat dimensions, teat-end condition, and inter-teat spacing are well documented in dairy cattle, where teat conformation shifts measurably across lactations [43]. This interpretation is internally consistent with the significant increase in teat base circumference reported here, since both reflect progressive remodeling of teat tissue rather than a change in the genetically fixed udder blueprint. By contrast, the predominance of the bowl-shaped udder (65.9%) and of moderate milk-vein development across all groups, with no significant age or number of foalings effect, is consistent with these being conserved, breed-characteristic conformation traits [22,28]; the absence of a milk-vein effect also argues against any large age-related change in mammary blood-flow demand in this uniformly managed herd.

The absence of any significant effect of age or number of foalings on individual milkings or on daily yield is, in our view, the expected consequence of the study design interacting with equine mammary physiology rather than evidence that these factors are biologically irrelevant. Because every mare was sampled in the same lactation month, the single largest source of variation in mare milk yield—stage of lactation, which drives the well-characterized rise-and-decline lactation curve [11,16]—was removed by design, leaving only the comparatively subtle age and number of foalings contrasts to be detected against large residual variation. The uniform extensive management on a single natural pasture further compressed environmental variation [45]. Superimposed on this is a physiological ceiling on harvestable milk: the equine udder stores most of its milk in the alveolar compartment, and its removal depends on oxytocin-mediated milk ejection triggered by suckling, so the standardized protocol of brief foal suckling followed by hand milking at fixed two-hour intervals inevitably harvested only a fraction of secreted milk—a fraction constrained by ejection efficiency and the small cisternal compartment, with fat-rich residual milk retained in the gland [10,17,44]. The large individual variability we observed—standard deviations frequently approaching half the group means, with daily yields around 3.4–4.5 L—together with modest subgroup sizes, further limited statistical power to resolve age and number of foalings effects. The literature is itself divided: some studies report that older, multiparous mares yield more than young primiparous mares [46], whereas others find number of foalings effects to be weak or inconsistent [12], and it is plausible that Kushum mares, as a breed selected over generations for dairy use under taboon conditions [34], reach mature yield early and maintain a stable plateau across most of their productive life. The stable daily yields we recorded are therefore consistent with a robust, early-maturing dairy phenotype rather than with an absence of biological signal.

In contrast to yield, the significant and coherent shifts in the minor, osmotically active solids of milk are physiologically interpretable and internally consistent. Lactose is the principal osmoregulator of milk volume and the dominant contributor to freezing-point depression, because its synthesis in the Golgi—catalyzed by the lactose synthase complex, in which α-lactalbumin modifies β-1,4-galactosyltransferase so that it accepts glucose as substrate—osmotically draws water into milk and largely sets its final volume and freezing point [47]. This framework parsimoniously explains the coupling we observed among lactose, freezing point, and density: milk with higher lactose (highest in the youngest, lowest-parity mares) had a more negative freezing point and higher density, whereas the intermediate group, with the lowest lactose and solids-not-fat, showed the least negative freezing point and the lowest density. The opposite behavior of free glucose, which increased with age and number of foalings even as lactose fell, is particularly informative, because free glucose is the immediate precursor pool for lactose synthesis; a reduced capacity to convert glucose to lactose—through lower α-lactalbumin or lactose-synthase activity, or reduced mammary glucose utilization in older mares—would be expected to leave a larger residual pool of unconverted free glucose while lowering lactose output [47]. The pronounced difference in milk urea, the single largest compositional contrast (approximately 61 mg/dL in the youngest, lowest-parity mares versus about 43 mg/dL in older groups), is best read as an index of protein–energy balance and nitrogen metabolism rather than of any udder trait, since milk urea tracks dietary protein relative to fermentable energy and the animal’s protein turnover; in dairy cattle, milk urea nitrogen in the 8–16 mg/dL range corresponds to dietary non-fiber-carbohydrate-to-crude-protein ratios of roughly 2.15–3.60 [48]. On a common pasture, the higher milk urea of young mares may reflect higher relative intake, greater protein turnover associated with ongoing somatic maturation, or differences in renal and hepatic nitrogen handling; because all animals grazed the same sward, a dietary-imbalance explanation is less likely than a physiological one. It should be noted that the absolute urea values in these mares exceed typical bovine reference ranges, so cow-derived thresholds cannot be transferred directly to equids, and our interpretation is necessarily comparative. Finally, the decreases in titratable acidity and lactic acid with age and number of foalings are most plausibly consequences of the lower solids and buffering capacity of the milk—protein, phosphate, and citrate determine the natural (apparent) acidity of fresh milk—rather than of microbial spoilage, given that all samples were freshly obtained and analyzed. These patterns are broadly consistent with our earlier reports on Kazakh mare milk [13,14,29] and with the compositional ranges published for other breeds [31,32].

The stability of fat, protein, and casein across age and number of foalings groups is equally interpretable and does not weaken the study. Mare milk fat is intrinsically low and exceptionally variable; here it ranged from roughly 0.2% to 0.7% with very large standard deviations, far below the fat content of cow milk and reflecting the general observation that equine milk fat is low relative to bovine and human milk [6]. This variability is rooted in the physiology of milk-fat secretion. Fat is the most variable milk constituent within a single milking, rising steeply as milking proceeds and being most concentrated in the fat-rich residual milk that is retained when ejection is incomplete [49]. In mares, where oxytocin release under a suckling-plus-hand-milking regimen is often insufficient to empty the alveoli, a large and inconsistent fraction of high-fat residual milk remains in the gland from one harvest to the next [17,30,44]. Under our fractional harvesting protocol, fat is therefore dominated by within-milking and residual-milk effects that swamp any age or number of foalings signal at this sample size, rendering the factor effectively unresolvable rather than truly absent. Protein and casein, by contrast, are under tighter genetic and homeostatic control and depend far more on lactation stage—held constant here—than on age or number of foalings [11,16]; the narrow ranges we recorded for protein (about 1.83–1.88%) and casein (about 1.29–1.34%), and the characteristically near-equal casein-to-whey balance of equine milk, in which whey proteins account for roughly 40% of total protein, unlike the casein-dominant profile of cow milk [1,5], are consistent with the absence of significant age and number of foalings effects and with the tightly controlled compositional architecture of horse milk. Several previous mare studies likewise report little or no number of foalings effect on protein and casein while detecting effects on carbohydrate and mineral fractions, in agreement with our findings [14,18,32,50].

A further limitation is that physicochemical composition was determined from equal-volume pooled samples rather than from individual milkings or milk-yield-weighted daily composites; therefore, within-day variation in milk composition could not be evaluated. An important limitation concerns the sample size available for udder morphometric analysis. Udder measurements were obtained from small numbers of mares, and classification resulted in markedly unbalanced groups, including only three mares in the youngest age group and two mares in the G1 number-of-foalings group. Such small subgroup sizes limit the estimation of within-group variability and reduce the statistical power and robustness of ANOVA and Tukey post hoc comparisons. Therefore, the observed differences in udder morphometric traits should be considered exploratory and interpreted cautiously until confirmed in larger and more balanced populations.

Several limitations of the present study should be considered when interpreting the results. First, mare age and number of foalings are biologically related characteristics, and their close relationship in this observational population limits the ability to distinguish their independent associations with morphometric and milk-related traits. For this reason, age and number of foalings were evaluated as separate classification factors, and the observed associations should not be interpreted as independent causal effects. Second, udder morphometric measurements were available for only 30 mares, resulting in small and markedly unbalanced subgroups, including groups with only two or three animals. Consequently, estimates for these subgroups have limited precision, and the corresponding inferential comparisons should be regarded as exploratory. Third, milk yield was measured under practical field conditions during five daytime milkings on only two consecutive days. Therefore, the reported daily milk yield represents harvestable milk obtained during the defined daytime milking period rather than total 24-h physiological milk production. The short observation period also limits inference regarding longer-term lactation performance. These considerations restrict the generalizability of the findings and warrant confirmation in larger, more balanced populations monitored over longer periods.

## 5. Conclusions

In conclusion, mare age and number of foalings were associated with specific phenotypic and milk characteristics in Kushum mares. After FDR correction, cannon bone circumference, body weight, udder length, and udder width differed significantly among both age and number-of-foalings groups, while teat shape was the only qualitative udder characteristic showing significant group-associated differences. The two-day mean daily milk yield did not differ significantly among age or number-of-foalings groups, although significant group × day interactions indicated differences in short-term milk-yield patterns between the two observation days. Physicochemical characteristics of pooled milk samples also differed among groups, particularly for SNF, lactose, glucose, freezing point, acidity, lactic acid, density, and urea. These findings indicate that mare age and reproductive history should be considered when characterizing the productive and phenotypic traits of Kushum mares.

## Figures and Tables

**Table 1:** Body and udder morphometric characteristics of Kushum mares according to mare age.

Parameter	4–7 yr	8–11 yr	12–16 yr	*p*-Value	FDR q
Height at withers	151.36 ± 1.50	149.83 ± 0.90	149.40 ± 1.27	0.520	0.693
Oblique body length	157.64 ± 1.70	155.00 ± 2.09	152.33 ± 1.64	0.111	0.167
Chest girth	182.64 ± 2.26	181.83 ± 1.68	181.40 ± 1.94	0.902	0.902
Cannon bone circumference	19.04 ± 0.21	20.12 ± 0.16	19.15 ± 0.19	<0.001	0.003
Body weight	489.93 ± 16.59	450.83 ± 11.13	431.27 ± 9.77	0.007	0.022
Udder length	26.33 ± 1.20	24.17 ± 1.02	19.60 ± 0.57	<0.001	0.002
Udder width	20.33 ± 0.33	19.17 ± 0.66	16.53 ± 0.32	<0.001	0.002
Udder depth	17.33 ± 0.88	16.67 ± 1.23	14.07 ± 0.46	0.061	0.105
Udder base circumference	43.67 ± 0.88	43.75 ± 1.22	44.47 ± 0.73	0.841	0.902
Teat length	4.90 ± 0.10	4.79 ± 0.29	4.60 ± 0.11	0.702	0.842
Teat base circumference	8.17 ± 0.44	9.70 ± 0.28	10.17 ± 0.31	0.023	0.056
Distance between teats	7.33 ± 0.88	7.25 ± 0.24	6.40 ± 0.21	0.046	0.092

**Table 2:** Body and udder morphometric characteristics of Kushum mares according to number of foalings.

Parameter	G1 (1–3)	G2 (4–7)	G3 (8–11)	*p*-Value	FDR q
Height at withers	151.85 ± 1.54	149.36 ± 0.85	149.50 ± 1.35	0.316	0.421
Oblique body length	158.23 ± 1.72	153.79 ± 1.99	153.00 ± 1.61	0.102	0.153
Chest girth	183.23 ± 2.35	181.71 ± 1.57	181.00 ± 2.04	0.728	0.728
Cannon bone circumference	19.05 ± 0.22	19.92 ± 0.20	19.19 ± 0.20	0.010	0.031
Body weight	496.46 ± 16.48	444.64 ± 10.37	432.79 ± 10.36	0.002	0.013
Udder length	27.50 ± 0.50	23.64 ± 1.01	19.79 ± 0.57	<0.001	0.011
Udder width	20.50 ± 0.50	18.93 ± 0.64	16.64 ± 0.33	0.003	0.013
Udder depth	17.50 ± 1.50	16.50 ± 1.06	14.07 ± 0.50	0.087	0.150
Udder base circumference	43.50 ± 1.50	43.64 ± 1.05	44.64 ± 0.76	0.715	0.728
Teat length	5.00 ± 0.00	4.80 ± 0.25	4.57 ± 0.12	0.588	0.706
Teat base circumference	8.50 ± 0.50	9.49 ± 0.29	10.25 ± 0.32	0.068	0.135
Distance between teats	7.50 ± 1.50	7.21 ± 0.21	6.36 ± 0.22	0.035	0.083

Data are presented as mean ± standard error. G1, G2, and G3 age groups correspond to 4–7, 8–11, and 12–16 years, respectively. Number of foalings groups correspond to 1–3, 4–7, and 8–11 foalings, respectively. Raw *p*-values are from one-way ANOVA; q-values are Benjamini–Hochberg FDR-adjusted across the 12 morphometric traits. Tukey pairwise comparisons were used for significant omnibus tests.

**Table 3:** Distribution of qualitative udder characteristics according to mare age and number of foalings.

Trait/Category	4–7 yr	8–11 yr	12–16 yr	Age *p*	Age q	G1 (1–3)	G2 (4–7)	G3 (8–11)	Foalings *p*	Foalings q
Udder shape: bath-shaped	4 (28.6)	5 (41.7)	1 (6.7)	0.107	0.161	4 (30.8)	5 (35.7)	1 (7.1)	0.171	0.257
Udder shape: bowl-shaped	10 (71.4)	6 (50.0)	11 (73.3)	0.107	0.161	9 (69.2)	8 (57.1)	10 (71.4)	0.171	0.257
Udder shape: round	0 (0.0)	1 (8.3)	3 (20.0)	0.107	0.161	0 (0.0)	1 (7.1)	3 (21.4)	0.171	0.257
Teat shape: cylindrical	7 (50.0)	1 (8.3)	1 (6.7)	0.010	0.030	7 (53.8)	1 (7.1)	1 (7.1)	0.005	0.015
Teat shape: conical	7 (50.0)	11 (91.7)	14 (93.3)	0.010	0.030	6 (46.2)	13 (92.9)	13 (92.9)	0.005	0.015
Milk veins: poor	3 (21.4)	3 (25.0)	1 (6.7)	0.628	0.628	3 (23.1)	3 (21.4)	1 (7.1)	0.694	0.694
Milk veins: moderate	7 (50.0)	6 (50.0)	11 (73.3)	0.628	0.628	6 (46.2)	8 (57.1)	10 (71.4)	0.694	0.694
Milk veins: well developed	4 (28.6)	3 (25.0)	3 (20.0)	0.628	0.628	4 (30.8)	3 (21.4)	3 (21.4)	0.694	0.694

Values are presented as n (%). Exact *p*-values are from the Fisher–Freeman–Halton test; q-values are Benjamini–Hochberg FDR-adjusted across the three qualitative udder traits within each classification factor.

**Table 4:** Daily milk yield of Kushum mares during two consecutive observation days.

Classification	Group	Day 1 (L/day)	Day 2 (L/day)	Two-Day Mean (L/day)
Age	4–7 yr	3.90 ± 0.41	4.63 ± 0.50	4.26 ± 0.45
	8–11 yr	3.44 ± 0.54	3.30 ± 0.51	3.37 ± 0.52
	12–16 yr	3.93 ± 0.55	3.81 ± 0.52	3.87 ± 0.53
Number of foalings	G1 (1–3)	4.08 ± 0.40	4.84 ± 0.49	4.46 ± 0.44
	G2 (4–7)	3.23 ± 0.48	3.13 ± 0.45	3.18 ± 0.46
	G3 (8–11)	4.05 ± 0.57	3.91 ± 0.55	3.98 ± 0.56

Values are mean ± SE. The five milkings performed each day were summed to obtain daily yield. The two-day mean was compared among groups by one-way ANOVA (age: *p* = 0.483; number of foalings: *p* = 0.195). Repeated-measures mixed models detected significant group × day interactions for both classifications (*p* < 0.001); therefore, day-specific patterns are reported descriptively and interpreted cautiously.

**Table 5:** Milk physicochemical composition according to mare age in Kushum mares.

Parameter	4–7 yr	8–11 yr	12–16 yr	*p*-Value	FDR q
Fat (%)	0.40 ± 0.06	0.78 ± 0.33	0.22 ± 0.04	0.088	0.136
Protein (%)	1.83 ± 0.05	1.83 ± 0.11	1.79 ± 0.06	0.888	0.984
SNF (%)	9.77 ± 0.05 ^a^	8.88 ± 0.32 ^b^	9.16 ± 0.19 ^ab^	0.015	0.035
TS (%)	10.26 ± 0.10	9.66 ± 0.46	9.52 ± 0.19	0.139	0.194
Lactose (%)	7.14 ± 0.05 ^a^	6.37 ± 0.25 ^b^	6.69 ± 0.11 ^ab^	0.005	0.017
Galactose (%)	0.352 ± 0.012	0.343 ± 0.010	0.354 ± 0.011	0.788	0.984
Glucose (%)	0.275 ± 0.010 ^a^	0.287 ± 0.017 ^ab^	0.333 ± 0.014 ^b^	0.010	0.028
Freezing point (°C)	−0.789 ± 0.007 ^a^	−0.703 ± 0.026 ^b^	−0.732 ± 0.012 ^b^	0.002	0.016
Acidity (°D)	10.11 ± 0.21 ^a^	9.75 ± 0.21 ^ab^	9.34 ± 0.15 ^b^	0.020	0.035
Lactic acid (%)	0.101 ± 0.002 ^a^	0.097 ± 0.002 ^ab^	0.093 ± 0.002 ^b^	0.020	0.035
Density (g/L)	1042.65 ± 0.32 ^a^	1038.26 ± 1.31 ^b^	1039.65 ± 0.74 ^b^	0.004	0.017
Citric acid (%)	0.141 ± 0.004	0.140 ± 0.009	0.139 ± 0.005	0.985	0.985
Urea (mg/dL)	61.48 ± 2.57 ^a^	43.34 ± 3.17 ^b^	42.94 ± 1.37 ^b^	<0.001	<0.001
Casein (%)	1.29 ± 0.03	1.31 ± 0.08	1.28 ± 0.04	0.914	0.984

Values are presented as mean ± SE. Different superscript letters within the same grouping factor indicate significant differences according to Tukey’s HSD test (*p* < 0.05). Different superscript letters indicate significant Tukey-adjusted pairwise differences. q-values are Benjamini–Hochberg FDR-adjusted across the 14 physicochemical traits.

**Table 6:** Milk physicochemical composition according to number of foalings in Kushum mares.

Parameter	G1 (1–3)	G2 (4–7)	G3 (8–11)	*p*-Value	FDR q
Fat (%)	0.40 ± 0.06	0.73 ± 0.30	0.23 ± 0.04	0.139	0.195
Protein (%)	1.83 ± 0.05	1.83 ± 0.10	1.79 ± 0.06	0.891	0.953
SNF (%)	9.77 ± 0.05 ^a^	8.95 ± 0.30 ^b^	9.11 ± 0.20 ^ab^	0.020	0.035
TS (%)	10.26 ± 0.10	9.69 ± 0.42	9.48 ± 0.20	0.128	0.195
Lactose (%)	7.14 ± 0.05 ^a^	6.43 ± 0.24 ^b^	6.66 ± 0.11 ^ab^	0.009	0.024
Galactose (%)	0.352 ± 0.012	0.343 ± 0.009	0.355 ± 0.012	0.743	0.946
Glucose (%)	0.275 ± 0.010 ^a^	0.285 ± 0.016 ^a^	0.337 ± 0.014 ^b^	0.005	0.019
Freezing point (°C)	−0.789 ± 0.007 ^a^	−0.711 ± 0.025 ^b^	−0.727 ± 0.012 ^b^	0.004	0.019
Acidity (°D)	10.11 ± 0.21 ^a^	9.75 ± 0.19 ^ab^	9.32 ± 0.16 ^b^	0.017	0.035
Lactic acid (%)	0.101 ± 0.002 ^a^	0.097 ± 0.002 ^ab^	0.093 ± 0.002 ^b^	0.017	0.035
Density (g/L)	1042.65 ± 0.32 ^a^	1038.60 ± 1.26 ^b^	1039.44 ± 0.76 ^b^	0.005	0.019
Citric acid (%)	0.141 ± 0.004	0.138 ± 0.008	0.141 ± 0.005	0.953	0.953
Urea (mg/dL)	61.48 ± 2.57 ^a^	43.21 ± 2.92 ^b^	43.04 ± 1.47 ^b^	<0.001	<0.001
Casein (%)	1.29 ± 0.03	1.31 ± 0.07	1.27 ± 0.04	0.890	0.953

Values are presented as mean ± SE. Different superscript letters indicate significant Tukey-adjusted pairwise differences. q-values are Benjamini–Hochberg FDR-adjusted across the 14 physicochemical traits.

## Data Availability

The original contributions presented in this study are included in the article/Supplementary Material. Further inquiries can be directed to the corresponding author.

## Supplementary Materials

The following supporting information can be downloaded at: https://www.mdpi.com/article/10.3390/ani16182864/s1. Figure S1: Schematic illustration of the anatomical landmarks and measurement procedures used for body and udder morphometric assessment in Kushum mares.

## Abbreviations

The following abbreviations are used in this manuscript:

ANOVA	Analysis of Variance
FTIR	Fourier Transform Infrared
HSD	Honestly Significant Difference
PCA	Principal Component Analysis
SNF	Solids-Not-Fat
TS	Total Solids
