# Supplementary material for: Effects of Mare Age and Number of Foalings on Body Morphometry, Udder Morphology, Milk Yield, and Milk Composition in Kushum Mares
Source: Animals (Basel). 2026 Sep 11;16(18):2864. doi: 10.3390/ani16182864 (PMC13603575; doi:10.3390/ani16182864)
Supplement: Supplementary file 1 [file animals-16-02864-s001.zip › animals-4517255-supplementary.pdf]

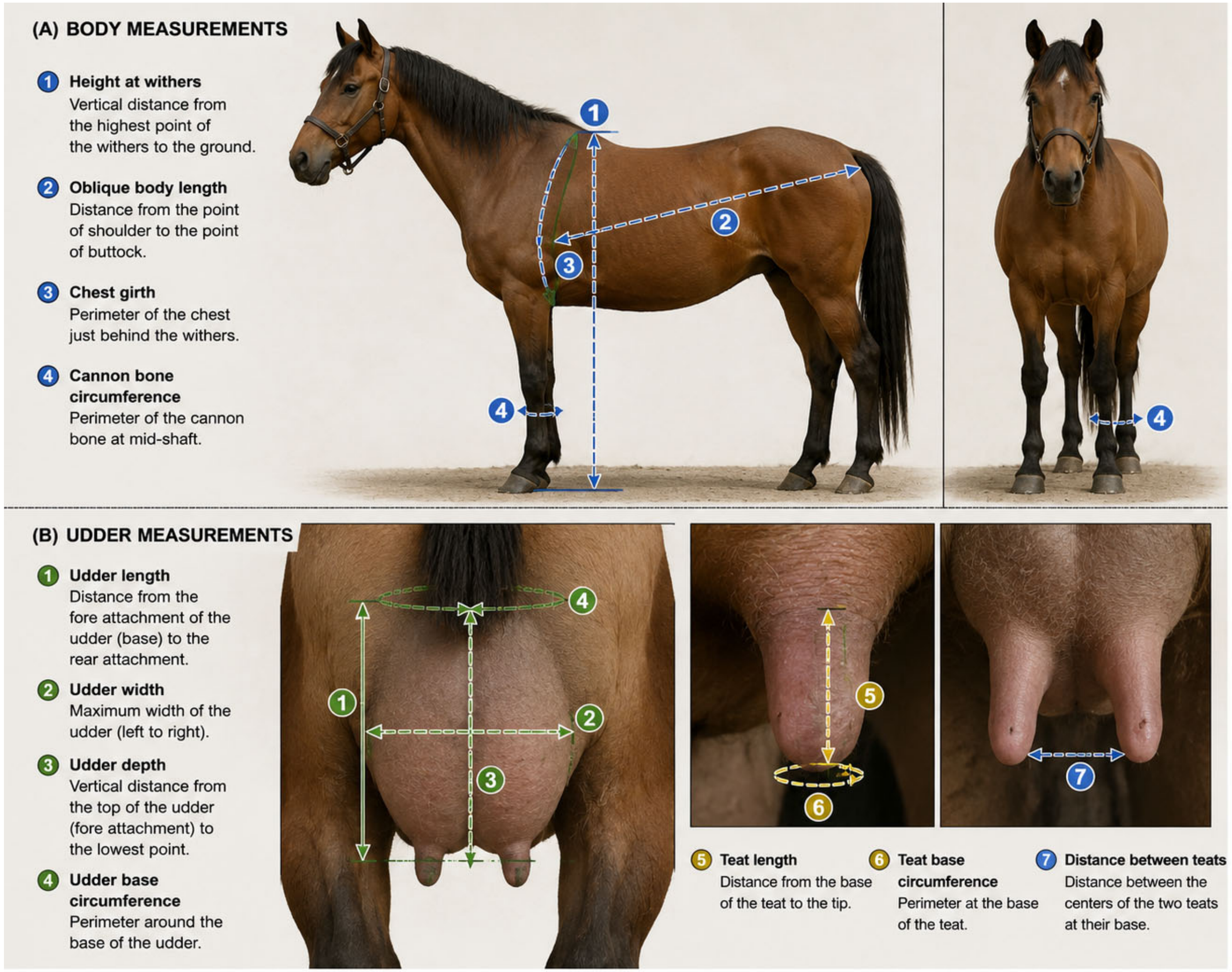

Figure S1. Schematic illustration of the anatomical landmarks and measurement procedures used for body and udder morphometric assessment in Kushum mares.
